# Host immune genetic variations influence the risk of developing acute myeloid leukaemia: results from the NuCLEAR consortium

**DOI:** 10.1038/s41408-020-00341-y

**Published:** 2020-07-16

**Authors:** J. M. Sánchez-Maldonado, D. Campa, J. Springer, J. Badiola, Y. Niazi, A. Moñiz-Díez, F. Hernández-Mohedo, P. González-Sierra, R. Ter Horst, A. Macauda, S. Brezina, C. Cunha, M. Lackner, M. A. López-Nevot, L. Fianchi, L. Pagano, E. López-Fernández, L. Potenza, M. Luppi, L. Moratalla, J. J. Rodríguez-Sevilla, J. E. Fonseca, M. Tormo, C. Solano, E. Clavero, A. Romero, Y. Li, C. Lass-Flörl, H. Einsele, L. Vazquez, J. Loeffler, K. Hemminki, A. Carvalho, M. G. Netea, A. Gsur, C. Dumontet, F. Canzian, A. Försti, M. Jurado, J. Sainz

**Affiliations:** 1 Genomic Oncology Area, GENYO, Centre for Genomics and Oncological Research: Pfizer / University of Granada / Andalusian Regional Government, PTS Granada, Granada, Spain; 2 Hematology department, Virgen de las Nieves University Hospital, Granada, Spain; 3 Instituto de Investigación Biosanitaria de Granada (ibs.Granada), Complejo Hospitales Universitarios de Granada/Universidad de Granada, Granada, Spain; 4 Department of Genetics, University of Pisa, Pisa, Italy; 5 Universitätsklinikum Würzburg, Medizinische Klinik II, Würzburg, Germany; 6 Division of Molecular Genetic Epidemiology, German Cancer Research Center (DKFZ), Heidelberg, Germany; 7 Hopp Children’s Cancer Center (KiTZ), Heidelberg, Germany; 8 Division of Pediatric Neurooncology, German Cancer Research Center (DKFZ), German Cancer Consortium (DKTK), Heidelberg, Germany; 9 Department of Internal Medicine and Radboud Center for Infectious Diseases, Radboud University Nijmegen Medical Center, Nijmegen, The Netherlands; 10 Genomic Epidemiology Group, German Cancer Research Center (DKFZ), Heidelberg, Germany; 11 Institute of Cancer Research, Department of Medicine I, Medical University of Vienna, Vienna, Austria; 12 Life and Health Sciences Research Institute (ICVS), School of Medicine, University of Minho, Braga, Portugal; 13 ICVS/3B’s - PT Government Associate Laboratory, Braga/Guimarães, Guimarães, Portugal; 14 Division of Hygiene and Medical Microbiology, Medical University of Innsbruck, Innsbruck, Austria; 15 Immunology department, Virgen de las Nieves University Hospital, Granada, Spain; 16 Istituto di Ematologia, Università Cattolica del S. Cuore, Rome, Italy; 17 Department of Medical and Surgical Sciences, University of Modena and Reggio Emilia, AOU Policlinico, Modena, Italy; 18 Hematology department, Hospital del Mar, Barcelona, Spain; 19 Rheumatology and Metabolic Bone Diseases department, Hospital de Santa Maria, CHLN, Lisbon, Portugal; 20 Rheumatology Research Unit, Instituto de Medicina Molecular, Faculty of Medicine, University of Lisbon, Lisbon Academic Medical Center, Lisbon, Portugal; 21 Hematology department, Hospital Clinico Universitario-INCLIVA, University of Valencia, Valencia, Spain; 22 Centre for Individualised Infection Medicine (CiiM) & TWINCORE, joint ventures between the Helmholtz-Centre for Infection Research (HZI) and the Hannover Medical School (MHH), Hannover, Germany; 23 Hematology department, University Hospital of Salamanca, Salamanca, Spain; 24 Division of Cancer Epidemiology, German Cancer Research Centre (DKFZ), 69120 Heidelberg, Germany; 25 Faculty of Medicine and Biomedical Center in Pilsen, Charles University in Prague, 30605 Pilsen, Czech Republic; 26 Department for Immunology & Metabolism, Life and Medical Sciences Institute (LIMES), University of Bonn, 53115 Bonn, Germany; 27 Université Claude Bernard Lyon I, Lyon, France; 28 Department of Medicine, University of Granada, Granada, Spain

**Keywords:** Risk factors, Genetics research

## Abstract

The purpose of this study was to conduct a two-stage case control association study including 654 acute myeloid leukaemia (AML) patients and 3477 controls ascertained through the NuCLEAR consortium to evaluate the effect of 27 immune-related single nucleotide polymorphisms (SNPs) on AML risk. In a pooled analysis of cohort studies, we found that carriers of the *IL13*_rs1295686A/A_ genotype had an increased risk of AML (*P*_Corr_ = 0.0144) whereas carriers of the *VEGFA*_rs25648T_ allele had a decreased risk of developing the disease (*P*_Corr_ = 0.00086). In addition, we found an association of the *IL8*_rs2227307_ SNP with a decreased risk of developing AML that remained marginally significant after multiple testing (*P*_Corr_ = 0.072). Functional experiments suggested that the effect of the *IL13*_rs1295686_ SNP on AML risk might be explained by its role in regulating IL1Ra secretion that modulates AML blast proliferation. Likewise, the protective effect of the *IL8*_rs2227307_ SNP might be mediated by TLR2-mediated immune responses that affect AML blast viability, proliferation and chemorresistance. Despite the potential interest of these results, additional functional studies are still warranted to unravel the mechanisms by which these variants modulate the risk of AML. These findings suggested that *IL13*, *VEGFA* and *IL8* SNPs play a role in modulating AML risk.

## Introduction

Acute Myeloid Leukaemia (AML) is a common haematological malignancy characterised by the clonal transformation of haematopoietic precursors that alter normal hematopoietic cell growth and differentiation^1^. Epidemiological studies suggested that AML onset can be triggered by multiple factors including age, sex, lifestyle, exposure to chemicals and a number of blood and congenital disorders^2^. However, the biological mechanisms underlying AML aetiology remain largely elusive. Even though cytogenetic analysis have allowed the stratification of AML patients into favourable, intermediate and unfavourable classes and has improved our ability to predict clonal evolution and disease progression^3^, many AML patients (~ 45%) have a normal karyotype, which suggests that additional genetic alterations are needed to develop the disease. Sequencing studies identified genes frequently mutated in AML, some of which predict poor prognosis (*NPM1*^wt^/*FLT3-ITD*^high^, *RUNX1*, *ASXL1* and *TP53*)^4^. Furthermore, it is increasingly evident that host immunity might also be implicated in AML risk and survival^5^. AML blasts activate immunosuppressive mechanisms to evade the immune system whereas immune response changes induced by the gut microbiota can also influence the anti-leukaemic effects of immune cells^6^. In addition, the efficacy of allogeneic stem cell transplantation (SCT) in eradicating AML is linked to the appearance of the graft-versus-leukaemia effect, mediated by the recognition of major histocompatibility antigens present in malignant blasts by T cells^7^. Likewise, the disappearance of these circulating T cells recognising AML or the loss of costimulatory (CD28/CD80, ICAM-1/CD11a) or inhibitory interactions (PD-1/PDL-1) eventually leads to relapse^8^ and the infusion of donor-derived CD8+ memory T cells induces remission in patients who relapsed following allogeneic SCT^9^. Considering that around two-thirds of AML patients relapse within the first 18 months after first-line therapy, clinical trials are trying to assess the efficacy of immunotherapies in AML and to unravel the interplay between the immune system and AML blasts. Considering the aspects detailed above, the purpose of this study was to conduct a two-stage case control association study including 654 AML patients and 3477 controls ascertained through the NuCLEAR consortium to evaluate whether 27 single nucleotide polymorphisms (SNPs) within the *IL4*, *IL8*, *IL8RB (CXCR2)*, *IL12A*, *IL12B*, *IL13*, *IFNG*, *IFNGR2*, *CCR5*, *MIF* and *VEGFA* loci influence the risk of developing AML. We also decided to investigate the correlation of selected SNPs with serum steroid hormone levels and their role in modulating immune responses after stimulation of whole blood, peripheral mononuclear cells (PBMCs) and macrophages with lipopolysaccharide (LPS), phytohemagglutinin (PHA), Pam3Cys and CpG.

## Material, subjects and methods

### Study design and study populations

We conducted a two-stage genetic association study to assess whether 27 functional single nucleotide polymorphisms (SNPs) within host immunity-related genes could influence AML risk. The discovery population consisted of 2027 European subjects (338 AML patients and 1689 healthy controls). AML patients were diagnosed by experienced clinicians and ascertained through the iNternational Consortium for LEukaemiA Research (NuCLEAR; Table 1). A set of AML patients were recruited from 2 Spanish medical institutions (Virgen de las Nieves University Hospital, Granada and Hospital of Salamanca, Salamanca), the University of Würzburg (Würzburg, Germany) and the University of Innsbruck (Innsbruck, Austria)^10^. Healthy controls included 667 Spanish blood donors from the REPAIR consortium^11^, 1000 German controls came from the Heinz-Nixdorf Recall (HNR) study^12^ and 22 donors of allogeneic stem cell transplantation from the Medical University of Innsbruck (Innsbruck, Austria). In accordance with the Declaration of Helsinki, all study participants provided their written informed consent to participate in the study and the ethical committees of all participating centres and hospitals approved the study.

**Table 1:** Demographic and clinical characteristics of AML patients and healthy controls.

Demographic characteristics	Discovery Population (*n* = 2027) 338 AML cases and 1689 healthy controls	Replication Population (*n* = 2104) 316 AML cases and 1788 healthy controls	Overall Population (*n* = 4131) 654 AML cases and 3477 healthy controls
AML cases			
Age (years)	55.19 ± 15.12	56.91 ± 17.25	56.02 ± 16.20
Sex ratio (male/female)	1.13 (179/159)	1.29 (178/138)	1.20 (356/297)
Country of origin			
Spain	257	97	354
Germany	26	74	100
Italy	–	145	145
Austria	55	–	55
Presentation			
de novo (*n*, %)	324 (95.86)	285 (90.19)	609 (93.12)
Secondary (*n*, %)	14 (04.14)	31 (09.81)	45 (06.88)
Healthy controls			
Age (years)*	56.10 ± 9.55	42.76 ± 11.76	49.57 ± 11.33
Sex ratio (male/female)	1.07 (871/818)	0.91 (848/937)	0.98 (1719/1754)
Country of origin			
Spain	667	507	1174
Germany	1000	1087	2087
Italy	–	194	194
Austria	22	–	22

Data are means ± standard deviation or percentage (%). A set of 99 patients (39 and 61 from the discovery and replication cohorts, respectively) could not be classified according to the FAB classification.

*AML* acute myeloid leukaemia

*Age was not available in a set of German controls included in the discovery (*n* = 1000) and replication cohorts (*n* = 1068).

### DNA extraction, SNP selection criteria and genotyping

Genomic DNA from all individuals was extracted from saliva or blood samples using the Oragen®-DNA Self-Collection kit (Oragene) or the Maxwell® 16 Blood DNA Purification kit (Promega) according to manufacturer’s instructions. SNP selection criteria were based on previous associations with haematological malignancies (AML, ALL, CML, CLL and non-Hodgkin lymphomas) or solid tumours and clinical related parameters (graft versus host disease, whole blood leucocyte counts, anthropometric measures, etc.) but also according to their functionality in Haploreg (https://pubs.broadinstitute.org/mammals/haploreg/haploreg.php), Regulome (https://www.regulomedb.org/regulome-search/), Blood eQTL browsers (https://genenetwork.nl/bloodeqtlbrowser/ and https://gtexportal.org/home/index.html), and linkage disequilibrium values (Table 2 and Supplementary Fig. 1). Genotyping was performed using KASP® probes (LGC Genomics, Hoddesdon, UK) according to previously reported protocols^13^. For quality control, ∼5% of DNA samples were randomly included as duplicates and concordance between duplicate samples was ≥99.0%. AML cases and controls were randomly distributed in 384-well plates and the person doing genotyping experiments did not know how AML cases and controls were distributed.

**Table 2:** List of selected markers.

Gene name	Gene symbol_SNP	dbSNP rs#	Risk allele	Reported associations with haematological malignancies, solid tumours, patient survival and different clinical parameters (GVHD, blood cell counts, BMI, etc.)	Refs.
*Interleukin 4 (IL4)*	*IL4_-1098*	rs2243248	G	Associated with increased risk of T-cell lymphomas and HBV reactivation in rituximab-treated patients with non-Hodgkin lymphoma (NHL)	^1,2^
	*IL4_IVS2-1443*	rs2243268	C	Associated with IL4 levels in whole blood and other tissues. Maps among promoter histone marks in bone marrow derived mesenchymal stem cells.	^3^
*Interleukin 8 (IL8)*	*IL8_-251*	rs4073	A	Associated with NHL risk and predictor of survival in follicular lymphoma. Associated with IL8 at both transcriptional and translational levels and with increased transmigration of primary neutrophils. Maps among promoter and enhancer histone marks in multiple primary immune cells, hematopoietic stem cells and bone marrow derived cultured mesenchymal stem cells. Regulome score 2b.	^4–7^
	*IL8_ IVS1* + *230 (+396)*	rs2227307	G	Associated with IL8 at both transcriptional and translational levels and with increased transmigration of primary neutrophils. Associated with follicular lymphoma patient survival	^6,7^
*CXC-Chemokine receptor 2 (IL8RB)*	*CXCR2_Ex3-1010*	rs1126580	G	Associated with CXCR2 and CXCR1 levels in whole blood (GTEx). Associated with shorter survival in diffuse large B-cell lymphoma and susceptibility to bile duct cancer	^8,9^
*Interleukin 12 alpha (IL12A)*	*IL12A_Ex7* + *277*	rs568408	A	Binding motifs for TFE and SIX5	
*Interleukin 12 beta (IL12B)*	*IL12B_Ex8* + *159 (+1188)*	rs3212227	C	Associated with risk of solid tumours and survival of follicular lymphoma patients	^5,10^
*Interleukin 13 (IL13)*	*IL13_-1069*	rs1800925	T	Associated with susceptibility to glioma, glioblastoma multiforme and CRC and an increased risk of leukopenia in metastatic renal cell carcinoma patients. Regulome score 2b	^11–13^
	*IL13_Ex4* + *98*	rs20541	T	Associated with susceptibility to multiple cancers including NHL, CRC and glioma. Associated with radiation-induced toxicity following treatment for non-small cell lung cancer. Regulome score 3a	^11,14–17^
	*IL13_IVS3-24*	rs1295686	A	HBV reactivation in rituximab-treated patients with NHL. Regulome score 3a	^2^
*Interferon gamma (IFN-γ)*	*IFNG_-1615*	rs2069705	C	Cytogenetic and molecular response with Imatinib in CML patients	^18^
	*IFNG_IVS3* + *284 (+2109)*	rs1861494	C	Cytogenetic and molecular response with Imatinib in CML patients	^18^
*Interferon gamma receptor 2 (IFN-γR2)*	*IFNGR2_Ex7-128*	rs1059293	T	Associated with Breast cancer risk. Regulome score 1f	^19^
	*IFNGR2_Ex2-16*	rs9808753	G	Associated with IFNGR2 levels in whole blood (GETx) and risk of NHL	^17^
*C-C chemokine receptor type 5 (CCR5)*	*CCR5_IVS1* + *246*	rs1799987	G	Associated with CCR2 levels in whole blood (GTEx) and with a more favourable MRD status in children with B-precursor acute lymphoblastic leukaemia (ALL). Regulome score 3a	^20^
	*CCR5_IVS1* + *151*	rs2734648	T	Associated with CCR1, CCR2 and CCR5 levels in whole blood (GTEx). Regulome score 3a	^21,22^
*Macrophage migration inhibitory factor (MIF)*	*MIF_-173*	rs755622	G	Associated with MIF and MIF-AS1 levels in whole blood (GTEx). Associated with solid and non-solid tumours such as childhood ALL. Maps near multiple promoter and enhancer histone marks in multiple tissues and all immune cell types and hematopoietic stem cells.	
*Vascular Endothelial Growth Factor alpha (VEGFα)*	*VEGFA_-2578*	rs699947	A	Associated with disease progression in chronic myeloid leukaemia (CML) and an increased risk of thyroid cancer and metastasis in men. Associated with survival in advanced-stage non-small-cell lung cancer. Regulome score 2b	^23–25^
	*VEGFA_-7*	rs25648	T	Associated with prognosis in AML and CLL patients. Associated with the risk of developing acute GVHD after allogeneic-stem cell transplantation. Associated with the risk of developing solid tumours such as bladder cancer and survival of patients with renal cell carcinoma. Maps near multiple promoter and enhancer histone marks in multiple tissues and all immune cell types and hematopoietic stem cells.	^26–29^
	*VEGFA_IVS2* + *1378*	rs3024994	T	Associated with a reduced risk of bladder cancer. Multiple promoter histone marks in immune cells and hematopoietic stem cells.	^26^
	*VEGFA_IVS7-919*	rs3025035	T	Associated with recurrence of hepatocellular carcinoma after transplantation and survival of patients with non-small cell lung cancer. Regulome score 3a	^30,31^
	*VEGFA_6112*	rs2146323	A	Alters a binding site for P53. Regulome score 2b	
	*VEGFA_IVS-99*	rs3024997	A	Associated with VEGFA mRNA expression in human monocytes	^32^
	*VEGFA_IVS7* + *763*	rs3025030	C	Maps near enhanced histone marks in 9 tissues	
	*VEGFA_5530*	rs998584	T	Associated with whole blood leukocyte count, adiponectin, HDL cholesterol and triglycerides levels. Associated with BMI and waist circumference	^33,34^
	*VEGFA_5958bp 3’of STP*	rs6899540	C	Maps near enhanced histone marks in multiple immune cell types including primary monocytes, primary B cells, NK cells, neutrophils, hematopoietic stem cells and bone marrow derived mesenchymal stem cells. Alters binding of 7 motifs (AP-1, BCL, Nkx2, Pax5…).	
	*VEGFA_6119bp 3’of STP*	rs6900017	T	Maps near enhanced histone marks in multiple immune cell types including primary monocytes, primary B cells, hematopoietic stem cells and bone marrow derived mesenchymal stem cells. Alters binding of 11 motifs (AP-1, p300, HDAC2, NFAT,…).	

eQTL data were gathered from the GTEx portal (https://gtexportal.org/home/) and Westra et al.^45^. Regulome score 1f (Eqtl + TF binding/DNase peak), 2b (TF binding+any motif+DNase Footprint+DNase peak) and 3a (TF binding+any motif+DNase peak) were considered as selection criteria. References are included as Supplementary Material.

*SNP* single nucleotide polymorphisms, *Allo-SCT* allogeneic stem cell transplantation, *OR* odds ratio, *CI* confidence interval, *NHL* non-Hodgkin lymphoma, *CML* chronic myeloid leukaemia, *CRC* colorectal cancer, *AIDS* acquired immune deficiency syndrome.

### Statistical analysis

Deviation from Hardy-Weinberg Equilibrium (HWE) was tested in the controls by chi-square (*χ*^2^). Logistic regression adjusted for sex and country of origin was used to assess the associations of the SNPs with AML risk assuming log-additive, dominant and recessive models. According the M_eff_ method^14^, 24 of 27 SNPs were independent and, consequently, the study-wide significant threshold was set to 0.0007 (0.05/24SNPs/3models). Statistical power was calculated using Quanto (v.12.4) assuming a log-additive model of inheritance.

### Replication cohort

For replication purposes, the most relevant findings (*P* < 0.05) were replicated in a cohort of 2104 subjects (316 AML cases and 1788 healthy controls). AML cases were recruited from an independent Spanish medical institution (Hospital General of Valencia, Valencia, Spain), from the University Hospital of Würzburg (Germany) and from two Italian medical institutions (Università Cattolica del Sacro Cuore, Rome and University of Modena and Reggio Emilia, AOU Policlinico, Modena) between 2015 and 2017. Five hundred and seven Spanish controls were blood donors recruited from the Blood Transfusion Centre (CRTS, Granada-Almería), 194 Italian controls from the REPAIR consortium, 1068 German controls from a second and independent set of the Heinz-Nixdorf Recall (HNR) study (University Hospital of Essen) and 19 donors of allogeneic stem cell transplantation from the University of Würzburg (Germany). The ethical committees of these centres approved the study.

### Functional analysis of the host immune-related variants

In order to determine the biological function of the most relevant SNPs, cytokine production in response to stimulation was measured in the 500 Functional Genomics cohort from the Human Functional Genomics Project (HFGP; http://www.humanfunctionalgenomics.org/). The Arnhem-Nijmegen Ethical Committee approved the study (42561.091.12) and biological specimens were collected after informed consent was obtained. We investigated whether any SNP was correlated with cytokine levels (IFNγ, IL1Ra, IL1β, IL6, IL8, IL10, TNFα, IL17, and IL22) after stimulation of peripheral blood mononuclear cells (PBMCs), whole blood or monocyte-derived macrophages from 408 healthy subjects with LPS (1 or 100 ng/ml), PHA (10 μg/ml), Pam3Cys (10 μg/ml), and CpG (100 ng/ml). After log transformation, linear regression analyses adjusted for age and sex were used to determine the correlation of selected SNPs with cytokine expression quantitative trait loci (cQTLs). All analyses were performed using R software (www.r-project.org/). In order to account for multiple comparisons, we used a significant threshold of 0.00006, i.e., the quotient of 0.05/(24 independent SNPs × 9 cytokines × 4 cell stimulants).

Detailed protocols for PBMCs isolation, macrophage differentiation and stimulation assays have been reported elsewhere^15–17^. Briefly, PBMCs were washed twice in saline and suspended in medium (RPMI 1640) supplemented with gentamicin (10 mg/ml), l-glutamine (10 mM) and pyruvate (10 mM). PBMC stimulations were performed with 5×10^5^ cells/well in round-bottom 96-wells plates (Greiner) for 24 h in the presence of 10% human pool serum at 37 °C and 5% CO_2_. Supernatants were collected and stored in −20 °C until used for ELISA. LPS (100 ng/ml), PHA (10μg/ml) and Pam3Cys (10 μg/ml) and CpG (100 ng/ml) were used as stimulators for 24 or 48 h. Whole blood stimulation experiments were conducted using 100 μl of heparin blood that was added to a 48 well plate and subsequently stimulated with 400 μl of LPS and PHA (final volume 500ul) for 48 h at 37 °C and 5% CO_2_. Supernatants were collected and stored in −20 °C until used for ELISA. Concentrations of human TNFα, IFNγ, IL1β, IL1RA, IL6, IL8, IL10, IL17, and IL22 were determined using specific commercial ELISA kits (PeliKine Compact, Amsterdam, or R&D Systems), in accordance with the manufacturer’s instructions.

### Correlation between steroid hormone levels and immunoregulatory SNPs

Given the impact of steroid hormones in modulating immune responses, we also evaluated the correlation of SNPs with serum levels of 7 steroid hormones (androstenedione, cortisol, 11-deoxy-cortisol, 17-hydroxy progesterone, progesterone, testosterone and 25 hydroxy vitamin D3) in a subset of subjects without hormonal replacement therapy or oral contraceptives (*n* = 280). Complete protocol details have been reported elsewhere^17^. Steroid hormones were analysed by liquid chromatography tandem–mass spectrometry (LC–MS) after protein precipitation and solid-phase extraction as described in Ter Horst et al.^17^ (see also Supplementary Material). Hormone levels and genotyping data were available for a total of 406 subjects. After log transformation, correlation between SNPs and serum steroid hormone levels was evaluated using linear regression adjusted for age and sex in R (http://www.r-project.org/). Significance thresholds were set to 0.0003 (0.05/24 independent SNPs/7 hormones).

## Results

This study was conducted in a discovery population comprised of 338 AML patients and 1689 healthy controls. AML patients had a similar age than controls (55.19±15.12 vs. 56.91±17.25) and showed a slightly increased male/female ratio compared to healthy controls (1.13 [179/159] vs. 1.07 [871/818]. Ninety five percent of the patients had *de novo* AML whereas the remaining 5% presented secondary disease evolving from a preceding dysplasia (Table 1).

The association analysis of the discovery population revealed that 11 immunoregulatory SNPs were associated with AML risk (*P* < 0.05; Table 3). We found that carriers of the *IFNGR2*_rs1059293T_ allele or the *IL4*_rs2243248G/G_, *IL13*_rs20541T/T_, *IL13*_rs1295686A/A_ and *VEGFA*_rs998584T/T_ genotypes showed an increased risk of developing the disease (OR_Dom_ = 1.51, *P* = 0.0074; OR_Rec_ = 4.33, *P* = 0.012; OR_Rec_ = 1.98, *P* = 0.028; OR_Rec_ = 2.16, *P* = 0.012; and OR_Rec_ = 1.40, *P* = 0.034). In addition, we observed that each copy of the *IL4*_rs2243268C_ allele was associated with a 1.31-fold increased risk of AML (OR_Add_ = 1.31, *P* = 0.042). On the other hand, we found that carriers of the *IL8*_rs2227307G_ and *VEGFA*_rs25648T_ alleles had a significantly decreased risk of AML (OR_Dom_ = 0.70, *P* = 0.012 and OR_Dom_ = 0.42, *P* = 0.00002) whereas each copy of the *IL8*_rs4073A_, *CCR5*_rs1799987G_, *CCR5*_rs2734648T_ alleles was associated with ~ 20–25% decreased risk of AML (OR_Add_ = 0.81, *P* = 0.020; OR_Add_ = 0.82, *P* = 0.043 and OR_Add_ = 0.75, *P* = 0.0044). Even though only the association of the *VEGFA*_rs25648_ SNP with a decreased risk of developing AML remained significant after correction for multiple testing in the discovery cohort (*P*_Corr_ = 0.0014), we found that the association of *IL8*_rs2227307_ and *IL13*_rs1295686_ with AML risk was confirmed in the replication population (OR_Dom_ = 0.74, *P* = 0.040 and OR_Dom_ = 2.24, *P* = 0.0051, respectively; Table 3). The pooled analysis including 4131 subjects (654 AML cases and 3477 controls) confirmed that carriers of the *IL13*_rs1295686_ genotype had a significantly increased risk of AML (OR_Rec_ = 2.18, *P* = 0.0002, *P*_Corr_ = 0.0144) whereas carriers of the *IL8*_rs2227307G_ allele had a decreased risk of developing the disease that remained marginally significant after correction for multiple testing (OR_Dom_ = 0.72, *P* = 0.0010, *P*_Corr_ = 0.072). Interestingly, although it was not statistically significant in the replication population likely due to the relatively limited power, the pooled analysis also revealed a strong association of the *VEGFA*_rs25648T_ allele with a decreased risk of AML that largely surpassed the stringent study-wide significant threshold (OR_Dom_ = 0.60, *P* = 0.0000012, *P*_Corr_ = 0.00086; Table 3).

**Table 3:** Association of immunoregulatory SNPs and risk of developing acute myeloid leukaemia.

Gene name	dbSNP rs#	Gene symbol_SNP	Risk allele	Discovery Population (*n* = 2027) 338 AML cases and 1689 healthy controls		*P*_Corr_	Replication Population (*n* = 2104) 316 AML cases and 1788 healthy controls		Overall Population (*n* = 4131) 654 AML cases and 3477 healthy controls		*P*_Corr_
				OR (95% CI)^a^	*P*		OR (95% CI)^a^	*P*	OR (95% CI)^a^	*P*	
*IL4*	rs2243248	*IL4_-1098*	G	**4.33 (1.37**–**13.7)**^b^	**0.012**	0.864	1.75 (0.31–9.67)^b^	0.52	**3.09 (1.26**–**7.58)**^b^	**0.014**	1.000
*IL4*	rs2243268	*IL4_IVS2-1443*	C	**1.31 (1.01**–**1.69)**	**0.042**	1.000	0.87 (0.66–1.15)	0.32	1.09 (0.90–1.31)	0.39	1.000
*IL8*	rs4073	*IL8_-251*	A	**0.81 (0.67–0.97)**	**0.020**	1.000	0.96 (0.76–1.20)	0.70	0.89 (0.78–1.01)	0.072	1.000
*IL8*	rs2227307	*IL8_ IVS1* + *230 (+396)*	G	**0.70 (0.53–0.92)**^c^	**0.012**	0.864	**0.74 (0.56–0.99)**^c^	**0.040**	**0.72 (0.59–0.87)**^c^	**0.0010**	0.072
*IL8RB*	rs1126580	*CXCR2_Ex3-1010*	A	0.82 (0.68–1.00)	0.044	1.000	1.05 (0.87–1.27)	0.61	0.95 (0.83–1.09)	0.49	1.000
*IL12A*	rs568408	*IL12A_Ex7* + *277*	A	2.48 (1.00–6.15)^b^	0.050	1.000	1.07 (0.35–3.32)^b^	0.90	1.66 (0.84–3.27)^b^	0.14	1.000
*IL12B*	rs3212227	*IL12B_Ex8* + *159 (+1188)*	C	0.99 (0.79–1.25)	0.96	1.000					
*IL13*	rs1800925	*IL13_-1069*	T	1.01 (0.80–1.28)	0.93	1.000					
*IL13*	rs20541	*IL13_Ex4* + *98*	T	**1.98 (1.08–3.65)**^b^	**0.028**	1.000	1.75 (0.90–3.39)^b^	0.10	**1.89 (1.21–2.94)**^b^	**0.0048**	0.346
*IL13*	rs1295686	*IL13_IVS3-24*	A	**2.16 (1.19–3.93)**^b^	**0.012**	0.864	**2.24 (1.27–3.93)**^b^	**0.0051**	**2.18 (1.45–3.26)**^b^	**0.0002**	**0.0144**
*IFNG*	rs2069705	*INFG_-1615*	C	1.10 (0.90–1.35)	0.34	1.000					
*IFNG*	rs1861494	*INFG_IVS3* + *284 (+2109)*	C	1.19 (0.96–1.47)	0.12	1.000					
*IFNGR2*	rs1059293	*INFGR2_Ex7-128*	T	**1.51 (1.11–2.05)**^c^	**0.0074**	0.533	0.90 (0.67–1.21)^c^	0.48	1.16 (0.94–1.43)^c^	0.16	1.000
*IFNGR2*	rs9808753	*INFGR2_Ex2-16*	G	1.05 (0.79–1.41)	0.73	1.000					
*CCR5*	rs1799987	*CCR5_IVS1* + *246*	G	**0.82 (0.67–0.99)**	**0.043**	1.000	0.98 (0.81–1.19)	0.85	0.90 (0.79–1.03)	0.13	1.000
*CCR5*	rs2734648	*CCR5_IVS1* + *151*	T	**0.75 (0.61–0.92)**	**0.0044**	0.317	1.12 (0.93–1.36)	0.24	0.93 (0.81–1.06)	0.27	1.000
*MIF*	rs755622	*MIF_-173*	G	0.88 (0.67–1.14)	0.32	1.000					
*VEGFA*	rs699947	*VEGFA_-2578*	A	1.05 (0.87–1.27)	0.58	1.000					
*VEGFA*	rs25648	*VEGFA_-7*	T	**0.42 (0.29–0.62)**^c^	**0.00002**	**0.0014**	0.79 (0.58–1.06)^c^	0.12	**0.60 (0.47–0.75)**^c^	**0.000012**	**0.00086**
*VEGFA*	rs3024994	*VEGFA_IVS2* + *1378*	T	0.87 (0.59–1.29)	0.49	1.000					
*VEGFA*	rs3025035	*VEGFA_IVS7-919*	T	1.08 (0.79–1.48)	0.62	1.000					
*VEGFA*	rs2146323	*VEGFA_6112*	A	1.01 (0.82–1.23)	0.95	1.000					
*VEGFA*	rs3024997	*VEGFA_IVS-99*	A	1.01 (0.83–1.24)	0.91	1.000					
*VEGFA*	rs3025030	*VEGFA_IVS7* + *763*	C	0.95 (0.72–1.26)	0.72	1.000					
*VEGFA*	rs998584	*VEGFA_5530*	T	**1.40 (1.03–1.89)**^b^	**0.034**	1.000	1.08 (0.80–1.47)^b^	0.61	1.24 (1.00–1.54)^b^	0.048	1.000
*VEGFA*	rs6899540	*VEGFA_5958bp 3’of STP*	C	1.01 (0.77–1.32)	0.93	1.000					
*VEGFA*	rs6900017	*VEGFA_6119bp 3’of STP*	T	0.94 (0.68–1.30)	0.72	1.000					

Association estimates were adjusted for sex and country of origin. *P* < 0.05 in bold. Corrected *P*-value was calculated by multiplying the unadjusted *P*-value by the number of tests performed (*n* = 72, 24 SNPs by 3 inheritance models tested).

*SNP* single nucleotide polymorphisms, *OR* odds ratio, *CI* confidence interval.

^a^Estimates were calculated according to an additive model of inheritance.

^b^Estimates were calculated according to a recessive model of inheritance.

^c^Estimates were calculated according to a dominant model of inheritance.

**Fig. 1:**
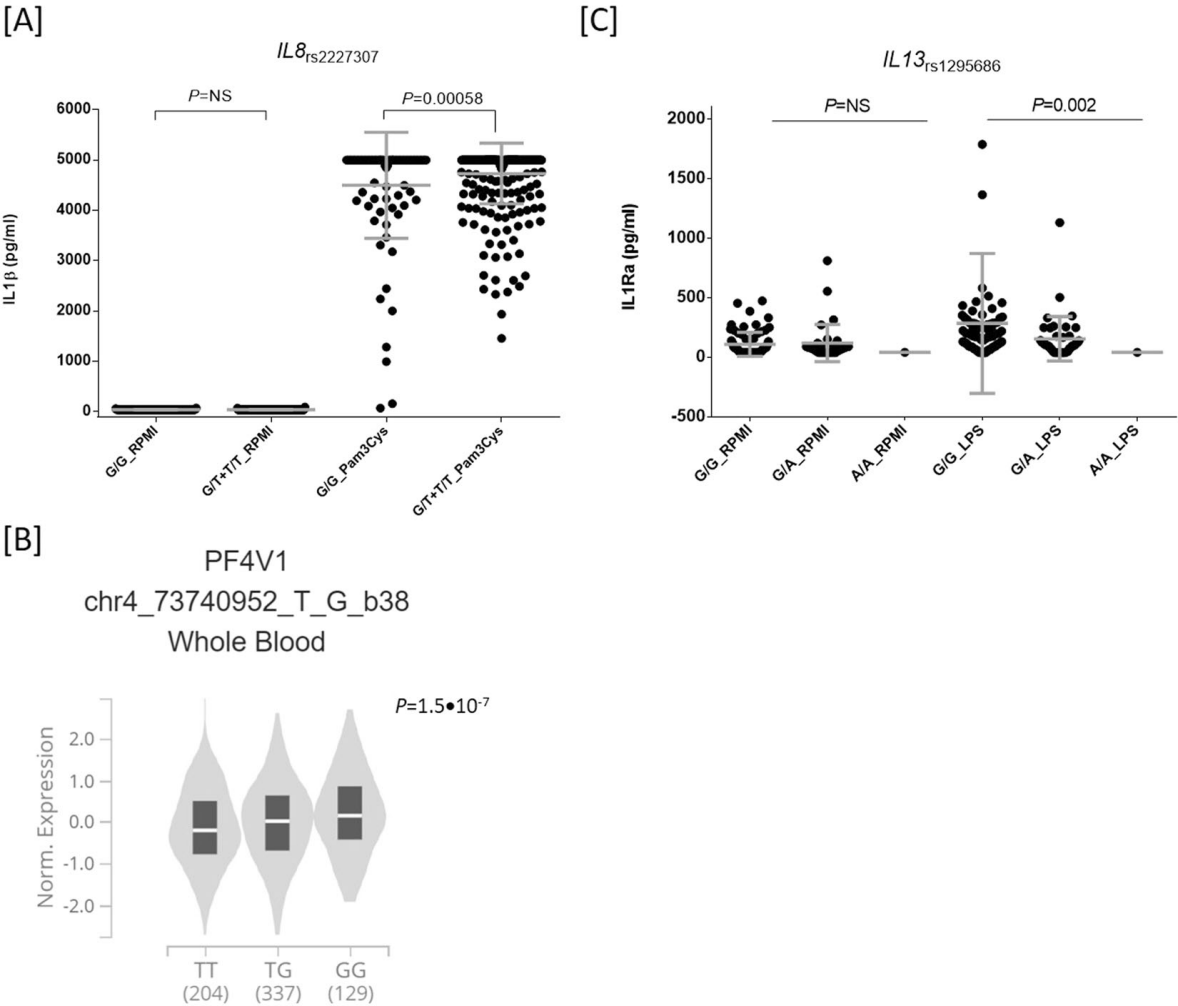
Functional impact of the *IL8*_rs2227307_ SNP on immune responses. Correlation between the *IL8*_rs2227307_ SNP and IL1β levels after stimulation of PBMCs (*n*=408) with Pam3Cys (10μg/ml) (**a**) or *PF4V* expression in peripheral blood (**b**) and correlation between the *IL13*_rs1295686_ SNP with IL1Ra levels after stimulation of PBMCs with LPS (100ng/ml) (**c**). Gene expression plot from the GTEx portal; https://gtexportal.org/home/index.html).

In an effort to determine the functional relevance of these polymorphisms, we performed in vitro stimulation experiments in a large cohort of healthy donors to investigate whether *IL8*, *IL13* and *VEGFA* SNPs could correlate with levels of IFNγ, IL1Ra, IL1β, IL6, IL8, IL10, TNFα, IL17, and IL22 after stimulation of PBMCs, whole blood or monocyte-derived macrophages with LPS, PHA, Pam3Cys, and CpG. These experimental studies revealed that carriers of the *IL8*_rs2227307T_ allele had increased levels of IL1β after the stimulation of PBMCs with Pam3Cys (*P* = 0.00058; Fig. 1a). Although this association did not survive multiple testing correction, these results suggested that this variant might have an impact on AML risk through the modulation of TLR2-immune responses. In support of a functional role of the *IL8*_rs2227307_ SNP in AML, it has been also reported that this SNP represents an eQTL for *PF4V (*Fig. 1b*)*, a locus involved in chemokine-mediated immune responses. Interestingly, although it neither reached statistical significance after multiple testing correction, we also found a negative correlation between the *IL13*_rs1295686A_ allele and levels of IL1Ra after stimulation of PBMCs with LPS (*P* = 0.002; Fig. 1c), which suggested that the *IL13* locus might play a role in the pathogenesis of AML likely through the modulation of IL1Ra-mediated immune responses. No correlation between selected SNPs and serum steroid hormone levels was found suggesting that the functional effect of these markers on the immune responses was not mediated by steroid hormones.

## Discussion

AML has been the object of investigations that have demonstrated that host immunity contributes to disease susceptibility. This study reports for the first time an association of the *IL13*_rs1295686_, *IL8*_rs2227307,_ and *VEGFA*_rs25648_ polymorphisms with AML risk. The association of the *IL13* and *VEGFA* SNPs with AML risk remained significant after multiple testing correction, whereas the association of *IL8*_rs2227307_ was not significant but close to the multiple testing significance threshold. These results suggested that the *IL13*, *VEGFA* and *IL8* loci might be susceptibility markers for AML.

The *IL13* gene is located on chromosome 5q31 and encodes for IL13, an immunoregulatory cytokine with pleiotropic functions. Several SNPs (rs20541, rs18000925 and rs1295686) within this gene have been consistently associated, at GWAS level, with immune-related diseases^18,19^ and haematological malignancies^20^. In this two-stage case control association study we found a consistent and statistically significant association of the *IL13*_rs1295686A/A_ genotype with an increased risk of developing AML that suggested a role of this locus in the pathogenesis of the disease. Mechanistically, we observed a negative correlation between the *IL13*_rs1295686A_ allele and IL1Ra levels after stimulation of PBMCs with LPS (*P* = 0.002; Fig. 1c). Although this association did not remain significant after correction for multiple testing, this finding supported our genetic results suggesting a role of the *IL13*_rs1295686_ SNP in the pathogenesis of AML. Considering our results but also those from an early report that demonstrated that IL1Ra levels are decreased in AML patients compared to controls^21^, we hypothesise that the effect of the *IL13*_rs1295686A_ allele on AML risk might be explained by its role in inhibiting IL1Ra secretion, likely through the inhibition of IL1Ra secretion from either AML blasts or healthy cells. In line with this argument, it has been consistently reported that IL1Ra inhibits AML blast proliferation^22^ and that it is associated with the immunosuppressive effect of the mesenchymal stem cells (MSCs) in the bone marrow that accounts for macrophage polarisation (toward the M2 phenotype) and B cell differentiation and survival^23^. Although at this point it is tempting to speculate that the *IL13*_rs1295686A_ allele, which correlates with lower levels of IL1Ra secretion, might represent a biomarker with a potential benefit in AML by antagonising IL1 effects on blast proliferation and blocking inflammation, we believe that additional functional experiments are still warranted to explain the exact mechanism by which the *IL13*_rs1295686_ variant influence the risk of AML.

Another interesting finding of this study was the consistent association of the *IL8*_rs2227307T_ allele with a decreased risk of developing AML. Although the association of the *IL8*_rs2227307_ SNP with AML risk remained only marginally significant after multiple testing correction, this finding suggested that the *IL8* locus might play a role in the pathogenesis of AML. The *IL8* gene is located on chromosome 4q12-q21 and encodes for IL8, a chemokine mainly produced by macrophages and epithelial cells. Previous studies have suggested that the blocking IL8-CXCR2 pathway might have a therapeutic potential in a variety of tumours^24–27^ including AML and myelodysplastic syndromes (MDS)^28^. However, the role of IL8 in AML is still scarce. A recent study has demonstrated that IL8 and its receptor are significantly overexpressed in AML and MDS patients^28^ and that the expression of these molecules also correlates with poor outcomes. In addition, it has been reported that the IL8-CXCR2 axis is highly expressed in hematopoietic stem cells and progenitor compartments in comparison with healthy controls^28^ and that this pathway plays a key role in the regulation of cancer stem cell function^29–31^ and mesenchymal stem cell-induced T cell proliferation. In addition, Schinke et al. (2015) have experimentally demonstrated that the inhibition of CXCR2 leads to decreased viability and clonogenic capacity of primary cells from AML patients, which pointed towards the use of IL8-CXCR2 pathway as novel therapeutic target^28^. In line with our genetic data and the notion of a role of the IL8 locus in the pathogenesis of AML, we found that carriers of the *IL8*_rs2227307T_ allele had increased levels of IL1β after the stimulation of PBMCs with Pam3Cys (*P* = 0.00058; Fig. 1a). These results suggested that the protective effect of the *IL8*_rs2227307_ SNP on AML risk might be mediated by TLR2-induced immune responses that are initially regulating IL1β secretion and, subsequently, IL8 production in a wide range of pathological conditions^32–35^. Given that the correlation of the *IL8*_rs2227307_ SNP with increased levels of IL1β did not reach the significance threshold after correction for multiple testing, we need to interpret these results with caution. Nonetheless, it worth mentioning that they were in agreement with previous studies showing that TLRs are expressed in multiple AML cell lines and primary AML samples^36^ and that stimulation of TLR2 in normal hematopoietic cells led to differentiation and proliferation of hematopoietic stem cells and myeloid progenitor cells. Furthermore, another study proposed a TLR2-binding cell-penetrating peptide as a promising candidate for targeted drug development in AML^37^. In addition to these findings, *IL8*_rs2227307_ has been also reported to be an eQTL for *PF4V (*Fig. 1b*)*, a locus involved in chemokine-mediated immune responses. These results suggest that the *IL8*_rs2227307_ polymorphism might also influence the risk of AML through chemotaxis stimulation in the microenvironment of the bone marrow (BM). In line with this notion, it has been demonstrated that IL8 is a hypoxia-regulated cytokine that promotes migration in mesenchymal stromal cells in the BM^38^ and that both endogenous and hypoxia-induced production of IL8 was higher in AML cases compared to controls and was prognostically unfavourable^38^. A more recent study has also suggested that IL8 blockade might be used as new therapeutic strategy for AML, as it prevents activated endothelial cell mediated proliferation and chemoresistance^39^.

Finally, even though we did not find any functional effect of the *VEGFA*_rs25648_ SNP to modulate immune responses, our genetic findings are in line with previous studies reporting an increased vascularity and VEGFA levels in AML patients, and a specific VEGFA-dependent vascular morphology in the leukemic BM^40^. In addition, it has been reported that VEGFA levels are an independent prognostic factor^41^ and that they modulate the appearance of graft versus host disease after SCT^42^. Based on the current evidence, we hypothesize that the *VEGFA*_rs25648_ SNP might influence the risk of developing AML through changes in BM vascularity and morphology and migration of human leukemia cells.

One of the major strengths of our study is the inclusion of two large populations. In the combined analysis, we had 80% power to detect an odds ratio of 1.33 (*α* = 0.0007) for a SNP with a frequency of 0.25, which underlined the feasibility of the study design. Another important strength of this study is the development of cytokine stimulation experiments and the measurement of seven serum steroid hormones in a large cohort of healthy subjects, which allowed us to investigate the functional role of the most relevant markers in modulating immune responses but also in determining serological steroid hormone levels. A drawback is the multicentric nature of this study that placed inevitable limitations such as the impossibility of uniformly collect cytogenetic and mutation profiles for a significant set of patients. Another limitation was that age was unknown for a subset of German controls. However, given that selected SNPs have not been linked to survival in AML, we think that age is not a modifying factor that could significantly influence the results.

In conclusion, we identified for the first time *IL8*, *IL13*, and *VEGFA* SNPs as susceptibility biomarkers for AML and provided new insights about the possible role of these loci in modulating innate and adaptive immune responses, and thereby becoming potentially clinical targets for enhancement of the antileukemic effects of immune cells.

Functional data used in this project have been meticulously catalogued and archived in the BBMRI-NL data infrastructure (https://hfgp. bbmri.nl/) using the MOLGENIS open source platform for scientific data^43^. This allows flexible data querying and download, including sufficiently rich metadata and interfaces for machine processing (R statistics, REST API) and using FAIR principles to optimise Findability, Accessibility, Interoperability and Reusability^44^.

## Supplementary information

Supplementary Material

Supplementary Figure 1
