# Supplementary material for: Host immune genetic variations influence the risk of developing acute myeloid leukaemia: results from the NuCLEAR consortium
Source: Blood Cancer J. 2020 Jul 16;10(7):75. doi: 10.1038/s41408-020-00341-y (PMC7366925; doi:10.1038/s41408-020-00341-y)
Supplement: Supplementary file 1 — Supplementary Material [file 41408_2020_341_MOESM1_ESM.docx]

**METHODS**

*Measurements of steroid hormones*

Cortisol, 11-deoxycortisol, androstenedione and 17-hydroxyprogesterone were analyzed by Liquid Chromatography Tandem-Mass Spectrometry (LCMSMS) after protein precipitation and solid-phase extraction as described in Ter Horst et al. (2016)[^35^](#_ENREF_35) with the following additional compound specific configurations and characteristics. Internal standard [^13^C_3_]-cortisol (Isoscience, King of Prussia, PA), ^2^H_5_-11-deoxycortisol (Isoscience, King of Prussia, PA), [^13^C_3_]- androstenedione (Isoscience, King of Prussia, PA) and [^13^C_3_]-17-hydroxyprogesterone (Isoscience, King of Prussia, PA) were used. Retention time was 1.46 min, 2.68 min, 3.70 min and 4.78 min for cortisol, 11-deoxycortisol, androstenedione and 17-hydroxyprogesterone respectively. An 9-point calibration curve was used cortisol (Sigma); 11-deoxycortisol (Sigma); androstenedione (Sigma) and 17-hydroxyprogesterone (Sigma). Two transitions (qualitative and quantitative) were monitored. Transitions (Q1>Q3) were m/z 363.4 > 121.1 (25kEV) and m/z 363.4 > 97.1 (34 kEV) for cortisol; m/z 366.4 > 124.1 (25 kEV) and m/z 366.4 > 100.1 (35 kEV) for ^13^C_3_-cortisol; m/z 347.2 > 97.1 (29 kEV) and m/z 347.2 > 109.1 (31 kEV) for 11-deoxycortisol; m/z 352.3 > 100.1 (31 kEV) and m/z 352.3 > 113.1 (29 kEV) for ^2^H_5_-11-deoxycortisol; m/z 387.2 > 97.1 (23 kEV) and m/z 387.2 > 109.1 (26 kEV) for androstenedione; m/z 290.2 > 100.1 (21 kEV) and m/z 290.2 > 112.1 (26 kEV) for ^13^C_3_- androstenedione; m/z 331.3 > 97.1 (31 kEV) and m/z 331.3 > 109.1 (31 kEV) for 17-hydroxyprogesterone; m/z 334.3 > 100.1 (30 kEV) and m/z 334.3 > 112.1 (33 kEV) for ^13^C_3_-17-hydroxyprogesterone. Dwell time was 100 ms, 40 ms, 100 ms and 60 ms for cortisol, 11-deoxycortisol, androstenedione and 17-hydroxyprogesterone, respectively. The method was linear assessed by CLSI EP6 protocol. Recovery was within 96.5 – 102% cortisol, 98.4 – 104% 11-deoxycortisol, 99.4 – 99.8% androstenedione and 98.6 – 102% 17-hydroxyprogesterone. Total CV for cortisol is 3,6% at 301 nmol/L and 3,1% at 1092 nmol/L. Total CV for 11-deoxycortisol is 5,9% at 2,1 nmol/L and 5,1% at 27 nmol/L. Total CV for androstenedione is 4,7% at 3,2 nmol/L and 4,1% at 23 nmol/L. Total CV for 17-hydroxyprogesterone is 5,6% at 2,6 nmol/L and 5,1% at 95 nmol/L. LOQ was 1.91 nmol/L (13.4% CV), 0.10 nmol/L (10% CV), 0.05 nmol/L (10% CV) and 0.10 nmol/L (10% CV) for cortisol, 11-deoxycortisol, androstenedione and 17-hydroxyprogesterone respectively.

**SUPPLEMENTARY MATERIAL**

**Supplementary Figure 1.** LD blocks reconstructed from genotype data generated.

**
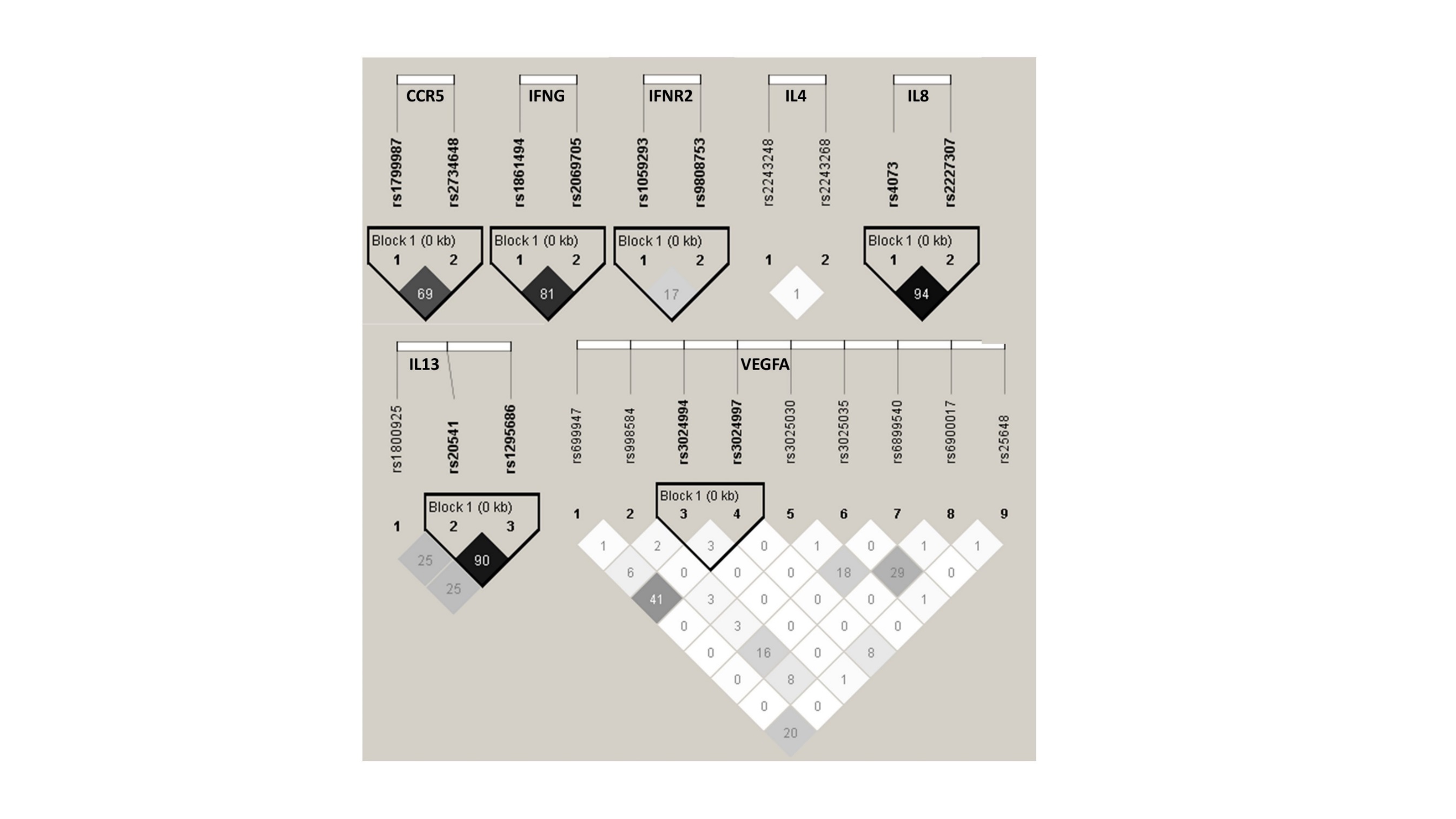
**

LD blocks reconstructed according to Gabriel et al. (Haploview).

LD values displayed as r^2^.

**REFERENCES (TABLE 2)**

1. Lan Q, Zheng T, Rothman N, Zhang Y, Wang SS, Shen M*, et al.* Cytokine polymorphisms in the Th1/Th2 pathway and susceptibility to non-Hodgkin lymphoma. Blood. 2006 May 15;107(10):4101-8.

2. Hsiao LT, Wang HY, Yang CF, Chiou TJ, Gau JP, Yu YB*, et al.* Human Cytokine Genetic Variants Associated With HBsAg Reverse Seroconversion in Rituximab-Treated Non-Hodgkin Lymphoma Patients. Medicine (Baltimore). 2016 Mar;95(11):e3064.

3. Westra HJ, Peters MJ, Esko T, Yaghootkar H, Schurmann C, Kettunen J*, et al.* Systematic identification of trans eQTLs as putative drivers of known disease associations. Nat Genet. 2013 Oct;45(10):1238-1243.

4. Colt JS, Rothman N, Severson RK, Hartge P, Cerhan JR, Chatterjee N*, et al.* Organochlorine exposure, immune gene variation, and risk of non-Hodgkin lymphoma. Blood. 2009 Feb 26;113(9):1899-905.

5. Cerhan JR, Wang S, Maurer MJ, Ansell SM, Geyer SM, Cozen W*, et al.* Prognostic significance of host immune gene polymorphisms in follicular lymphoma survival. Blood. 2007 Jun 15;109(12):5439-46.

6. Benakanakere MR, Finoti LS, Tanaka U, Grant GR, Scarel-Caminaga RM, Kinane DF. Investigation of the functional role of human Interleukin-8 gene haplotypes by CRISPR/Cas9 mediated genome editing. Sci Rep. 2016 Aug 8;6:31180.

7. Baecklund F, Foo JN, Bracci P, Darabi H, Karlsson R, Hjalgrim H*, et al.* A comprehensive evaluation of the role of genetic variation in follicular lymphoma survival. BMC Med Genet. 2014 Oct 8;15:113.

8. Habermann TM, Wang SS, Maurer MJ, Morton LM, Lynch CF, Ansell SM*, et al.* Host immune gene polymorphisms in combination with clinical and demographic factors predict late survival in diffuse large B-cell lymphoma patients in the pre-rituximab era. Blood. 2008 Oct 1;112(7):2694-702.

9. Hsing AW, Sakoda LC, Rashid A, Andreotti G, Chen J, Wang BS*, et al.* Variants in inflammation genes and the risk of biliary tract cancers and stones: a population-based study in China. Cancer Res. 2008 Aug 1;68(15):6442-52.

10. Zhou L, Yao F, Luan H, Wang Y, Dong X, Zhou W*, et al.* Functional polymorphisms in the interleukin-12 gene contribute to cancer risk: evidence from a meta-analysis of 18 case-control studies. Gene. 2012 Nov 15;510(1):71-7.

11. Su T, Mi Y, Zhang L, Wang S, Lu H, Shi L*, et al.* Association between IL13 gene polymorphisms and susceptibility to cancer: a meta-analysis. Gene. 2013 Feb 15;515(1):56-61.

12. Diekstra MH, Liu X, Swen JJ, Boven E, Castellano D, Gelderblom H*, et al.* Association of single nucleotide polymorphisms in IL8 and IL13 with sunitinib-induced toxicity in patients with metastatic renal cell carcinoma. Eur J Clin Pharmacol. 2015 Dec;71(12):1477-84.

13. Schwartzbaum J, Ahlbom A, Malmer B, Lonn S, Brookes AJ, Doss H*, et al.* Polymorphisms associated with asthma are inversely related to glioblastoma multiforme. Cancer Res. 2005 Jul 15;65(14):6459-65.

14. Wang SS, Carreon JD, Hanchard B, Chanock S, Hisada M. Common genetic variants and risk for non-Hodgkin lymphoma and adult T-cell lymphoma/leukemia in Jamaica. Int J Cancer. 2009 Sep 15;125(6):1479-82.

15. Sainz J, Rudolph A, Hoffmeister M, Frank B, Brenner H, Chang-Claude J*, et al.* Effect of type 2 diabetes predisposing genetic variants on colorectal cancer risk. J Clin Endocrinol Metab. 2012 May;97(5):E845-51.

16. Amirian E, Liu Y, Scheurer ME, El-Zein R, Gilbert MR, Bondy ML. Genetic variants in inflammation pathway genes and asthma in glioma susceptibility. Neuro Oncol. 2010 May;12(5):444-52.

17. Chen Y, Zheng T, Lan Q, Foss F, Kim C, Chen X*, et al.* Cytokine polymorphisms in Th1/Th2 pathway genes, body mass index, and risk of non-Hodgkin lymphoma. Blood. 2011 Jan 13;117(2):585-90.

18. Kim DH, Kong JH, Byeun JY, Jung CW, Xu W, Liu X*, et al.* The IFNG (IFN-gamma) genotype predicts cytogenetic and molecular response to imatinib therapy in chronic myeloid leukemia. Clin Cancer Res. 2010 Nov 1;16(21):5339-50.

19. Quan L, Gong Z, Yao S, Bandera EV, Zirpoli G, Hwang H*, et al.* Cytokine and cytokine receptor genes of the adaptive immune response are differentially associated with breast cancer risk in American women of African and European ancestry. Int J Cancer. 2014 Mar 15;134(6):1408-21.

20. Davies SM, Borowitz MJ, Rosner GL, Ritz K, Devidas M, Winick N*, et al.* Pharmacogenetics of minimal residual disease response in children with B-precursor acute lymphoblastic leukemia: a report from the Children's Oncology Group. Blood. 2008 Mar 15;111(6):2984-90.

21. Xue Y, Xu H, Rong L, Lu Q, Li J, Tong N*, et al.* The MIF -173G/C polymorphism and risk of childhood acute lymphoblastic leukemia in a Chinese population. Leuk Res. 2010 Oct;34(10):1282-6.

22. Wang CD, Li TM, Ren ZJ, Ji YL, Zhi LS. Contribution of Macrophage Migration Inhibitory Factor -173G/C Gene Polymorphism to the Risk of Cancer in Chinese Population. Asian Pac J Cancer Prev. 2015;16(11):4597-601.

23. Hsiao PJ, Lu MY, Chiang FY, Shin SJ, Tai YD, Juo SH. Vascular endothelial growth factor gene polymorphisms in thyroid cancer. J Endocrinol. 2007 Nov;195(2):265-70.

24. Masago K, Fujita S, Kim YH, Hatachi Y, Fukuhara A, Nagai H*, et al.* Effect of vascular endothelial growth factor polymorphisms on survival in advanced-stage non-small-cell lung cancer. Cancer Sci. 2009 Oct;100(10):1917-22.

25. Kim DH, Xu W, Kamel-Reid S, Liu X, Jung CW, Kim S*, et al.* Clinical relevance of vascular endothelial growth factor (VEGFA) and VEGF receptor (VEGFR2) gene polymorphism on the treatment outcome following imatinib therapy. Ann Oncol. 2010 Jun;21(6):1179-88.

26. Garcia-Closas M, Malats N, Real FX, Yeager M, Welch R, Silverman D*, et al.* Large-scale evaluation of candidate genes identifies associations between VEGF polymorphisms and bladder cancer risk. PLoS Genet. 2007 Feb 23;3(2):e29.

27. Kim DH, Lee NY, Lee MH, Sohn SK. Vascular endothelial growth factor gene polymorphisms may predict the risk of acute graft-versus-host disease following allogeneic transplantation: preventive effect of vascular endothelial growth factor gene on acute graft-versus-host disease. Biol Blood Marrow Transplant. 2008 Dec;14(12):1408-16.

28. Lozano-Santos C, Martinez-Velasquez J, Fernandez-Cuevas B, Polo N, Navarro B, Millan I*, et al.* Vascular endothelial growth factor A (VEGFA) gene polymorphisms have an impact on survival in a subgroup of indolent patients with chronic lymphocytic leukemia. PLoS One. 2014;9(6):e101063.

29. Dornbusch J, Walter M, Gottschalk A, Obaje A, Junker K, Ohlmann CH*, et al.* Evaluation of polymorphisms in angiogenesis-related genes as predictive and prognostic markers for sunitinib-treated metastatic renal cell carcinoma patients. J Cancer Res Clin Oncol. 2016 Jun;142(6):1171-82.

30. Wu LM, Xie HY, Zhou L, Yang Z, Zhang F, Zheng SS. A single nucleotide polymorphism in the vascular endothelial growth factor gene is associated with recurrence of hepatocellular carcinoma after transplantation. Arch Med Res. 2009 Oct;40(7):565-70.

31. de Mello RA, Ferreira M, Soares-Pires F, Costa S, Cunha J, Oliveira P*, et al.* The impact of polymorphic variations in the 5p15, 6p12, 6p21 and 15q25 Loci on the risk and prognosis of portuguese patients with non-small cell lung cancer. PLoS One. 2013;8(9):e72373.

32. Zeller T, Wild P, Szymczak S, Rotival M, Schillert A, Castagne R*, et al.* Genetics and beyond--the transcriptome of human monocytes and disease susceptibility. PLoS One. 2010 May 18;5(5):e10693.

33. Dastani Z, Hivert MF, Timpson N, Perry JR, Yuan X, Scott RA*, et al.* Novel loci for adiponectin levels and their influence on type 2 diabetes and metabolic traits: a multi-ethnic meta-analysis of 45,891 individuals. PLoS Genet. 2012;8(3):e1002607.

34. Willer CJ, Schmidt EM, Sengupta S, Peloso GM, Gustafsson S, Kanoni S*, et al.* Discovery and refinement of loci associated with lipid levels. Nat Genet. 2013 Nov;45(11):1274-1283.
