# Supplementary figures and images for: Host immune genetic variations influence the risk of developing acute myeloid leukaemia: results from the NuCLEAR consortium
Source: Blood Cancer J. 2020 Jul 16;10(7):75. doi: 10.1038/s41408-020-00341-y (PMC7366925; doi:10.1038/s41408-020-00341-y)

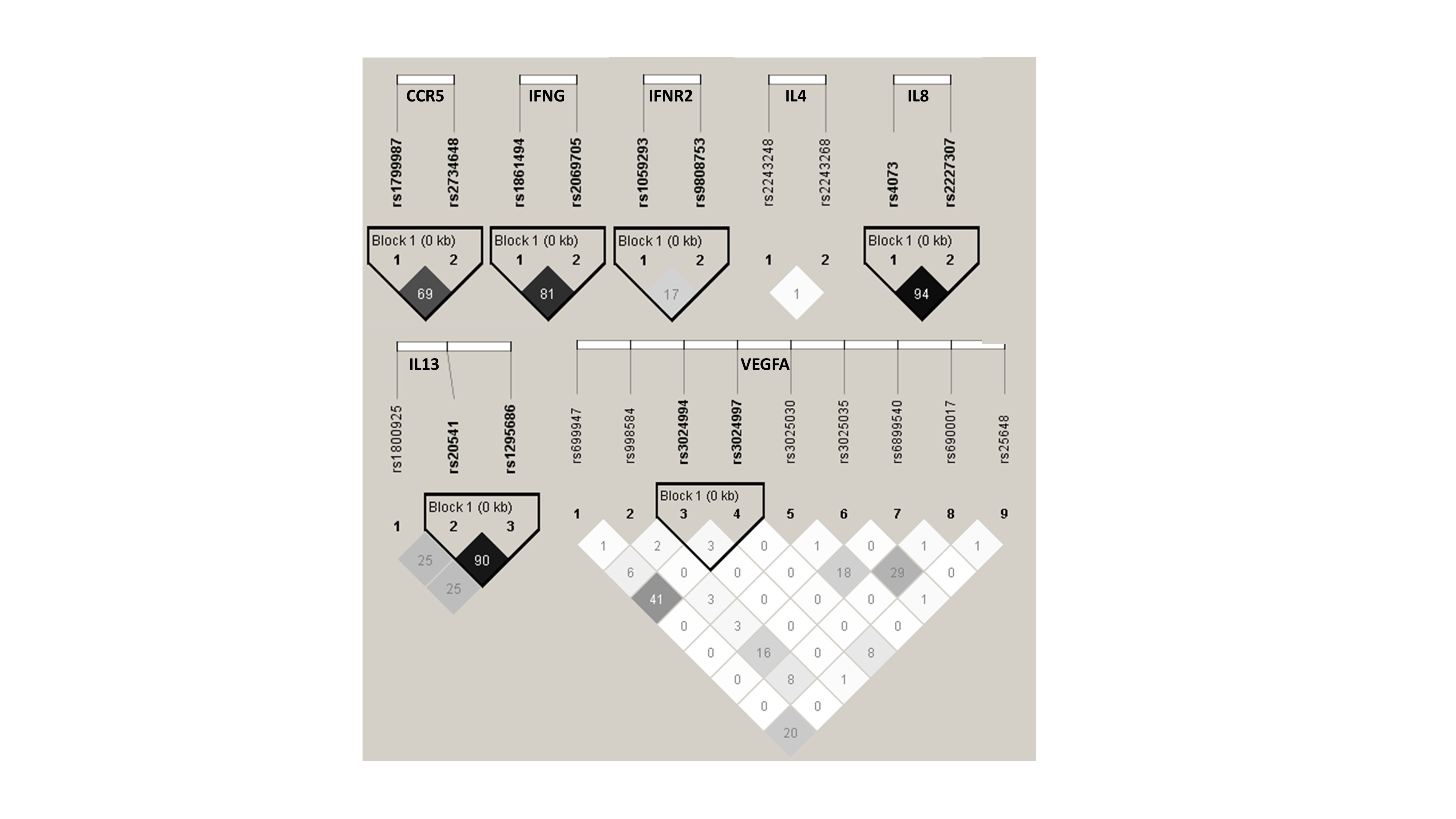

Supplement: Supplementary file 2 — Supplementary Figure 1 [file 41408_2020_341_MOESM2_ESM.jpg]
